# The impact of a rosemary containing drink on event-related potential neural markers of sustained attention

**DOI:** 10.1371/journal.pone.0286113

**Published:** 2023-06-01

**Authors:** Leigh Martin Riby, Sheridan Edwards, Heather McDonald, Mark Moss

**Affiliations:** 1 Psychology Department, Northumbria University, Newcastle-upon-Tyne, United Kingdom; 2 Institute of Psychiatry, Psychology and Neuroscience, King’s College London, London, United Kingdom; Murdoch University, AUSTRALIA

## Abstract

**Background:**

Research suggests that the ingestion or aroma of rosemary enhances cognitive ability in both rodents and humans. However, how rosemary facilitates cognition and the precise therapeutic impacts on information processing remains unclear.

**Hypothesis/purpose:**

This pilot study used the temporal precision of event-related potentials (ERPs) to examine the cognitive-enhancing benefits of a rosemary drink. Neural markers of sustained attention were used as indices to explore whether rosemary facilitates concentration in general or the allocation of resources to task-relevant information only.

**Study design and methods:**

In a between-subject design (rosemary vs water control drink), 48 adults performed a 3-stimulus visual oddball task. Participants differentiated between rare target stimuli (index of task-relevant attentional processes; P3b ERP) embedded in a train of frequent stimuli. The presentation of an infrequent novel stimulus was also included (index of task-irrelevant stimulus processing; P3a ERP). Throughout the session, electroencephalograms (EEG) were collected and time-locked to the presentation of the target (P3b) and novel (P3a) stimulus types.

**Results:**

The primary analyses revealed facilitation of the P3a in particular with a medium Cohen’s effect size reported. The investigation of the P3b component, although less reliable, also had a medium effect. The subsidiary consideration of the association between behaviour and the ERPs provided a further level of explanation regarding the therapeutic effect of rosemary on cognition. Indeed, the pattern of associations was suggestive of strategy differences during the performance of the task across the treatment group., although these data should be treated with caution.

**Conclusions:**

These pilot data provide critical insights into the utility of rosemary to facilitate different aspects of attention. In particular, data are consistent with rosemary providing additional attentional resources to enhance the processing of stimuli we encounter, irrespective of task relevance. Indeed, the enhancement of both P3a and P3b components following rosemary administration may indicate that the herb enhances the processing of all stimuli in the environment. We argue for the use of both behavioural and EEG methods to explore the therapeutic effects of herbal compounds.

## Introduction

The worldwide practice of herbal medicine has increased in popularity as an alternative and complementary medical technique mainly due to accumulated evidence derived using robust scientific methods and evaluation. Despite the increasing knowledge regarding the impact of herbs and herbal extracts, and the therapeutic benefits resulting from their use, there remains a limited understanding of the mechanisms behind the observed effects. Pharmacologically active compounds have been identified in several plant extracts and essential oils [1] and linked to enhancements of human cognition [2–4]. However, since there is a lack of characterization of the precise mechanisms of action and a limited understanding of the underlying neural processes and the specificity of the cognitive enhancement effects, further experimental work is warranted. With these issues in mind, we utilize the precision of brain event-related potential (ERPs; derived from electroencephalograms, EEG) methodology to examine the neuro-cognitive processes that guide our attention to stimuli in the environment that may profit from rosemary drink consumption.

In a related field, researchers have investigated the impact of the aromas of essential oils on EEG spectra. Chemosensory and olfactory processing components and EEG correlates have been catalogued elsewhere (e.g. [5]). However, relevant to the cognitive facilitation properties, Diego and colleagues influential work [6] employed converging behavioural and electrophysiological methods to track mood and cognitive effects of both lavender (sedative) and rosemary (stimulant) aromas. A different behavioural profile was observed, with relaxed and drowsy self-reports for the lavender compared to heightened alertness for those exposed to rosemary. EEG spectra revealed decreased Alpha and Beta brain activity for rosemary compared to increased Beta for the lavender experimental groups. The authors argued that the EEG spectral patterns mirrored the accompanying behavioural mood data. Given the difficulties in determining the functional significance of the EEG described and the more unambiguous characterization of this EEG spectra in recent years (e.g. [7] Marcuse et al.), we need to be cautious interpreting these data. Regardless, a different cognitive phase added a level of analysis to aid interpretation. During math calculation, lavender exposure gave rise to more accurate and faster responses during task completion. Interestingly, rosemary aroma’s apparent benefit in terms of increased self-reported alertness and EEG spectral changes had the subsequent effect of improving the speed of math computations. Sanders et al. [8] confirmed the idiosyncratic effects of these two aromas on EEG signals. They moreover noted asymmetric lateral shifts between the hemispheres that were also characteristic of and consistent with the purported behavioural impact of these aromas. Lateralization investigations of this nature point to the aroma of rosemary influencing valence (positive) and motivational (approach) behaviours subserved by increased left hemisphere activation.

Since the initial observations of EEG spectral changes, more comprehensive studies have utilized autonomic nervous system responses (ANS), including heart rate and blood pressure in their investigations. Sayorwan et al. [9] used both ANS and a self-report measure of arousal and confirmed the stimulating effects reported in earlier studies. The inclusion of EEG measurement corroborated this pattern. A switch from more dominant Alpha to Beta activations was consistent with enhanced alertness and cognition. Notably, the authors identified 1,8 cineole as a candidate driving the cognitive enhancing properties of rosemary. Moss and Oliver [10] provided direct support in their novel investigation of the mental impacts by incorporating simultaneous measurement of serum levels of 1, 8 –cineole. For the first time, the authors demonstrated significant relationships between blood serum indices in reaction time and accuracy on serial subtractions (attentional control and working memory) and rapid visual information-processing (vigilance and sustained attention) tasks. This study mirrored earlier work by Kovar et al. [11], who reported enhancement of locomotive activity in rodents accompanied by changes in 1, 8 –cineole levels in the blood irrespective of inhalation or oral administration of rosemary (indicative of direct brain action). Although findings point to 1,8 –cineole as a vital element that drives the facilitation effect via the cholinergic system [12], other essential mechanisms have been suggested [13].

The use of event-related potentials in psychopharmacological research is invaluable, given the precision tracking of cognitive processes possible during tasks. Chemosensory and olfactory related response research has dominated the field with detailed perceptual components outlined [14]. Polich and Criado [15] have described the P3a and P3b ERP components as useful neuro-psychological and pharmacological assessment tools in the realm of cognition. The current study will employ the 3-stimulus visual oddball task to generate these two neuro-markers. During the task, when participants attend to the target task-relevant stimuli, a positive peak is elicited at 300 to 500 ms over central-parietal scalp sites (P3b). Evidence suggests the P3b amplitude reflects the maintenance of a stimulus in working memory when the mental representation of the stimulus context needs updating [16]. Second, when participants process a distractor stimulus, an earlier deflection, shorter in duration, with a fronto-central distribution is elicited (P3a). The P3a depends on frontal lobe functioning and reflects the relatively automatic attention capture by rare task-irrelevant stimuli [17]. The choice of scalp sites for recording the P3a and P3b ERPs is based on the topographical separable distributions reflecting different underlying neural mechanisms. The P3a component is typically observed at the frontal-central scalp sites, with maximum amplitude at the Fz or Cz electrode. In contrast, the P3b component is typically observed at the parietal-central scalp sites, with maximum amplitude at the Cz or Pz electrode. These distributions have been extensively studied and reported in the literature, including the influential work by Polich and colleagues (e.g. [16]). The putative brain mechanism outlined by Polich and colleagues suggests frontal lobe and anterior cingulate cortex engagement while processing novel, unexpected stimuli and temporal-parietal activity associated with working memory while processing the relevant stimuli during the task. The neurotransmitter activity associated with the P300 generation is unclear (see [18]). However, the dual-transmitter hypothesis suggests the P3a frontal-attention processing is related to dopaminergic activity and consistent with the anatomical work, temporal-parietal norepinephrine involvement for the P3b ([16]; see also [19] for ERP neurobiology discussions). Although neglected in the area, one study has utilized the auditory P3 as a biomarker in investigating aroma ylang-ylang [20].

In addition to neurocognitive characterization, there are several sources of evidence that the P3a and P3b index related aspects of sustained attention (e.g. [21]). This proposition is significant given the evidence from behavioural work pinpointing the rosemary effect to aspects of sustained attention (The Rapid Visual Information Processing Task) and attentional control/working memory (The Serial Subtraction Task; [10]), and for that reason, the primary model adopted here. Given our knowledge of the P3a, and earlier behavioural work, we firstly predict that if rosemary has a positive impact on sustained attention, in general, it is anticipated that there will be an increase in the P3a amplitude. Specifically, rosemary would increase attentional resources and promote the information processing of all stimuli in the environment, irrespective of relevance. If this holds true, our findings would be consistent with previous pharmacological studies that have shown a general improvement in the attentional system, which is associated with an enhancement of both the P3a and P3b ERP components [22]. Unlike the finding of increased P3a in ‘deficit’ populations where distraction is prominent (e.g. ADHD; [23]), an increase in the P3a would only be seen in a positive light if no disruption to task-related information processing is observed (i.e. behavioural changes or a P3b reduction). Regarding the P3b, if rosemary impacts on the attention of task-relevant processing only, we predict the enhancement of the P3b component. Under this view, rosemary would only provide additional attentional resources to increase task focus. It is also possible that a decrease in the P3a may accompany the increase in the P3b if rosemary improves attention resources and attentional control, enabling the inhibition of task-irrelevant information. The oddball task was employed here so that we could focus on isolating and comparing the scalp activity across conditions. Due to the simplicity of the task we do not anticipate differences in behavioural performance.

## Methods

### Participants

Forty-eight adults based in the UK aged between 18–63 completed the study (32 women; Mean age = 30.7 SD = 11.2). There was no difference in age between the control (31.2) and rosemary conditions (30.2; p>0.75). All participants were right-handed and reported no history of neurological disorders. Participants were also screened for a current diagnosis of depression, anxiety or drug/alcohol abuse, suffering from migraines, or any of the following: anaemia, heart disorder, high blood pressure, respiratory disorder, diabetes, current pregnancy, history of seizures, or currently taking any prescribed, illicit or herbal drugs, and any food allergies or sensitivities. Participants were renumerated with a £10 Amazon voucher. The allocation of participants to the treatment was randomly assigned prior to the start of the study. The number of participants and trials described below were based on recommendations by Polich and colleagues (e.g. [24]) and Luck and colleagues (e.g. [25]) and also similar studies employing the P300 paradigm to investigate the impact of nutrient intake in a between subjects design (e.g. [26]).

### Materials

The three-stimulus oddball task has been used extensively in cognitive neuroscience to track attentional and memory processes [16]. This computerized task was presented using the E-Prime presentation software (pstnet.com) on a 17 ½-inch monitor. Participants were instructed to respond to the infrequent target stimuli and ignore all other stimuli. The target stimulus (green square, area = 16cmsq) appeared on 13% of trials in a train of a more frequent standard stimulus (red circle, area = 12.6cmsq) appearing on 74% of trials. An unexpected novel stimulus (blue square, area = 256cmsq) appeared on 13% of the trials. The study consisted of 4 blocks of 150 trials each. Stimuli remained on screen for 100ms, followed by an interstimulus interval between 830ms-930ms. Due to the simplicity of the task, reaction times and accuracy were collected for correct hits to the target only. Responses to the target stimuli were made on a computer keyboard spacebar. There was a 10-trial practice phase.

### Treatment

**Fig 1:**
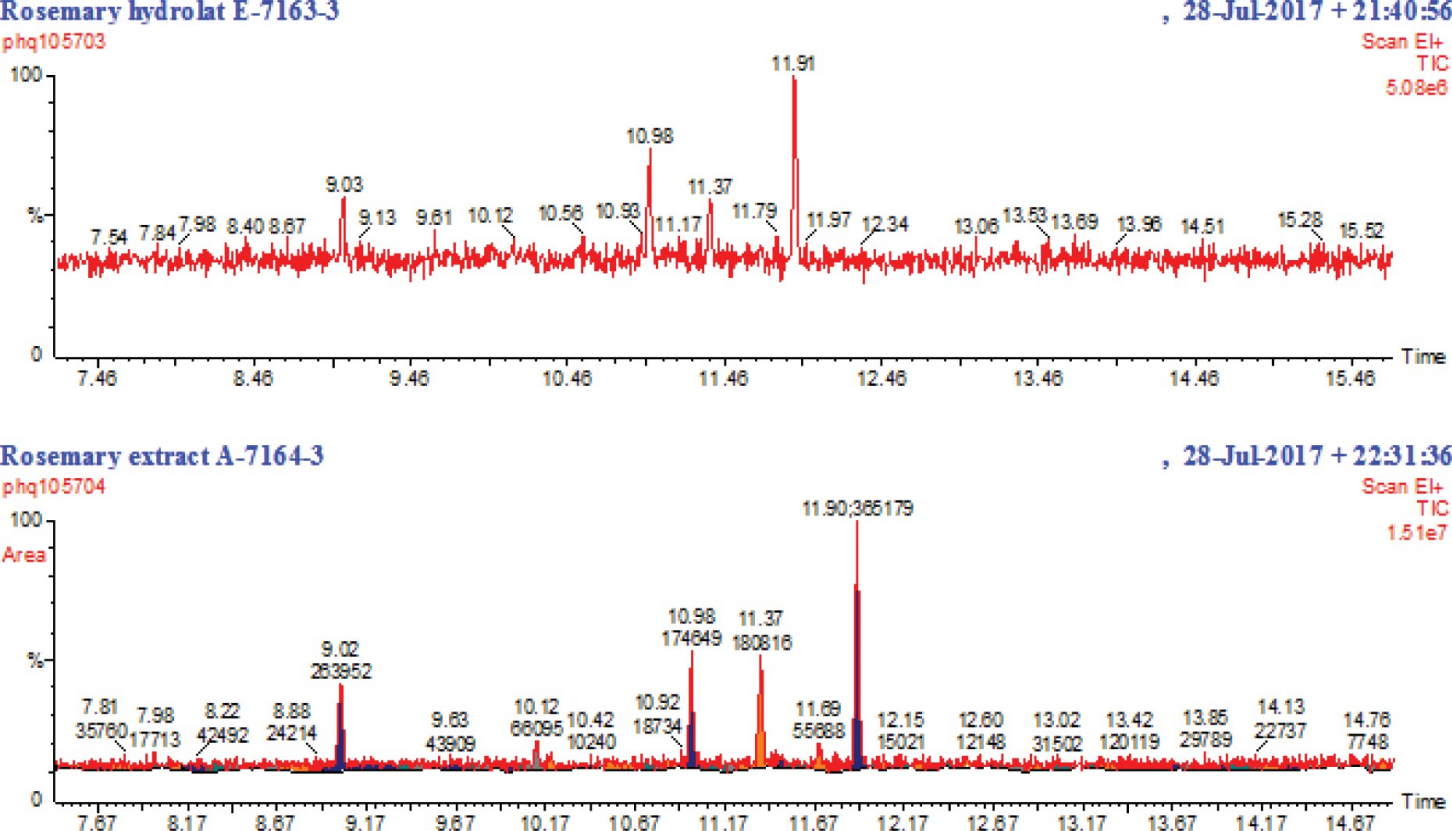
GCMS traces of trimethylsilyated samples for both the hydrolat (top) and extract (bottom) used to produce the shot strength rosemary water. 1,8-Cineole emerges at 9.03 min, camphor at 10.98 min, isoborneol at 11.37 min, and heptanaol at 11.90 min (matched using the National Institute of Standards and Technology (NIST) Spectral Database.

Rosemary water was supplied in 330ml bottles by No1. Bottanicals, Bischheim House, First Floor, 19–20 Berners Street, London, W1T 3NW. The water contains an extract of volatile oil soluble components extracted by steam distillation and a hydrolat of water soluble extracts captured into the distillate water of fresh rosemary. Production and analysis of the hydrolat and extract was undertaken by Blue Sky Botanics, Castle Farm, Upton Bishop, Ross-on-Wye, HR9 7UW. The two elements have different constituent profiles with the extract containing a number of terpenes predominantly 1,8-cineole (0.025mg/ml), and also Rosmarinic acid (0.13mg/ml). The hydrolat has substantially lower levels of terpenes, including 1,8-cineole (0.012 mg/ml), no Rosmarinic acid, but quinnic acid and glucosamine-like compounds are detectable. Gas Chromatography Mass Spectrometry (GCMS) traces are presented in Fig 1.

Rosemary water has a distinct taste. To blind participants as to condition, those assigned to the Rosemary water condition were told that a placebo drink with the same taste had been prepared, and those in the plain water condition were told that the rosemary extract had the taste components removed. All participants were told that the researcher did not know which treatment the participant was being given. Although double blinding was not carried out bias was minimized by the measures described and random allocation of the participants to the conditions.

### ERP recording

**Fig 2:**
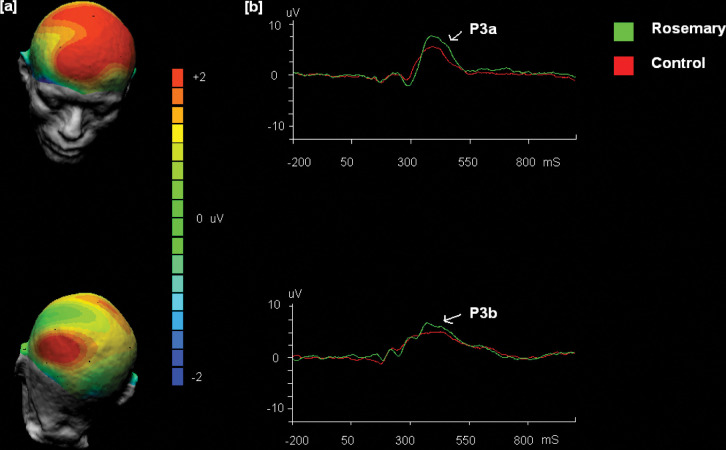
The grand average event-related potentials (ERPs) showing responses to novel and target stimuli across treatment group. **A**, The scalp distributions (rosemary minus control difference waves) of the P3a (350-470ms; average in window) and P3b ERP components (350-500ms; average in window) **b**, for clarity, mean amplitude (uV) for selected frontal (Fz; P3a) and central scalp (Cz; P3b) sites. The pre-stimulus of -200mS is also shown.

EEGs were recorded from a 32-channel electrode cap (Biosemi, Amsterdam) based on the international 10–20 system [27]. The montage included four midline scalp sites (Fz,Cz,Pz,Oz), 14 sites over the left hemisphere (FP1,AF3,F3,F7,FC1,FC5,C3,T7,CP1,CP5,P3,P7,PO3,O1), and 14 sites over the right hemisphere (FP2,AF4,F4,F8,FC2,FC6,C4,T8,CP2,CP6,P4,P8,PO4,O2). However, to capture the components of interest, the following electrodes were the focus (see [16] for discussion of the P300 spatial distributions). These were Frontal (Fz), Central (Cz) and Parietal (Pz,) scalp regions. Electrodes were placed above and below the right eye to record the electrooculogram artefacts. The CMS (Common Mode Sense electrode; placed centre between Cz and C3 electrodes) and DRL (Driven Right Leg electrode; placed between the Cz and C4 electrodes) were used as the “ground” electrodes (see Biosemi.com). The EEG recordings were referenced using the average of all electrodes method (see [25] for discussion). All signals were digitized at a rate of 2048Hz, band-pass-filtered at 0.46–30 Hz. Offline processing comprised of the following—. Automatic eyeblink correction (Ocular artefact reduction algorithms; [28]), artefact rejection (values outside the range -75uV to 75uV) and baseline corrected with the pre-stimulus interval (-200ms) using Neuroscan 4.3 Edit software (Compumedics, El Paso). Each participant recording was also screened manually for other artefacts we anticipated not being captured by the software or where the artefact was not detected adequately (e.g. eye movement, muscle activity). Epoching of the continuous EEG files were then carried out from stimulus triggers sent from the task presentation computer. The measurement intervals were selected based on visual inspection of the grand average ERPs (Fig 2B; P3a 350-470ms, P3b 350-500ms) and consideration of the time intervals reported in our previous research using this paradigm (e.g. [21]) and reported elsewhere (e.g. [16]). Average amplitudes were calculated in these ranges for the P3a and P3b. For an example of a similar approach to defining the components, see [29]. After eye blink correction and artefact rejection and removal of trials that did not make the rejection criteria there were on average 75.3% and 75.8% of available trials entered into the condition averages for the P3a and P3b, respectively. There were no differences in the trials entered into the averages across the treatment condition (P3a, p>0.29; P3b, p>0.31). Seven participants were excluded from the behavioral and EEG data analysis due to equipment errors and/or noisy data. Three participants had only behavioural data available, and one participant’s second session was unavailable due to equipment failure.

### Procedure

Ethical approval was obtained from the Department of Psychology Ethics Board at Northumbria University. The investigation was conducted according to the principles expressed in the Declaration of Helsinki. After reading an information sheet and providing written informed consent, testing commenced in the same quiet, moderately lit EEG laboratory between 9 am and 5 pm. Participants were given either 330ml of still water containing pure rosemary extract or ordinary still water (not containing the extract). Participants were randomly allocated to either the rosemary extract or ordinary still water (control) condition, which was prepared before the participants’ arrival. Participants were blind to condition allocation (see above Treatment Section). A 15-minute timer was set after the water was consumed to allow full absorption of the extract before the assessment was administered. During this time, the EEG equipment was set up using multi-purpose electrolyte gel (SignaGel®, Parker Labratories Inc., USA) with two additional electrodes placed above the eyebrow of the left eye and below the left eye on the upper cheek. Participants were asked to remain as relaxed as possible. The experimenter demonstrated to the participant the impact of excessive eye blinks, eye movements and muscular activity on the EEG signal. The 3-stimulus visual oddball task was then administered. Before beginning the experimental phase, participants were given a practice block for the oddball task. The study lasted approximately 25 minutes, after which a full debrief was provided.

## Results

### Analytical strategy

Although our focus was the ERP data, we first consider the behavioural performance for completeness. Independent t-tests analyzed the hit rate and reaction to hits with Cohen’s *d*, also calculated to establish the treatment effect sizes. The primary analyses considered group differences in the two subcomponents of the P300 (P3a vs. P3b) to examine the specificity of the rosemary effect regarding sustained attention and the processing of task-relevant target information (P3b) and task-irrelevant novel distracting information (P3a). That is, a series of mixed model analyses of variance (ANOVA) were carried out with the treatment (control vs. rosemary) as the between-subjects factor. The session (one vs. two) was the first within subjects factor. Session one data was the pooled data from block 1 and 2 of the oddball task. Session two data was the pooled data from blocks three and four of the oddball task. We incorporated this factor to increase the reliability of the data and examine possible habituation effects as the oddball task proceeded (see [30]). Location was the second within-subjects factor with 2 levels. The P3a has primarily been associated with frontal activity, novelty processing and task-irrelevant information processing and has consistently been associated with frontal-central scalp activation [16, 31]. Therefore, the location factor for the P3a was frontal Fz and central Cz locations. The topographically varied and functionally distinct P3b component associated with the attention to task-relevant information has scalp activity located at the parietal and central scalp regions (e.g. [16, 32]). Therefore, the two levels for the location factor were the Pz and Cz for this component. Cohen’s *d* values were also calculated between treatment for each location where the P3a and P3b are centred to allow easy comparison with other electrophysiological work in the area. Finally, a secondary correlation analysis was performed on the wider locations evident in Fig 2 (component mean amplitudes) and behavioural performance (reaction time and accuracy). Our reasoning here was to examine the ERPs (broader regions seen in Fig 2; see 29 for a similar approach) in relation to behaviour to add an additional level of explanation to aid in interpreting the data and, importantly, generate hypotheses for future experimental work.

The independent t-test analyses of the behavioural data revealed no significant effects (all p > .05) for Target stimuli Hits (Control 76%, SD 21.5 vs. Rosemary 78%, SD 14.7; *d* = 0.14) or Reaction Times to Hits (Control 274ms, SD 14.3 vs. Rosemary 278ms, SD9.1; *d* = 0.30). The main focus was the impact of treatment on the P3a and P3b electrophysiology during the Oddball task. A series of 2 (Group; Control, Rosemary) by 2 (Session; One, Two) by 2 (Location; Frontal, Central P3a; Central, Parietal P3b) ANOVAs were conducted. The analysis for the P3a component revealed a main effect of session, F (1, 36) = 17.69, p<0.001, a main effect of location, F (1, 36) = 32.13, p<0.001 and a main effect of treatment F (1,36) = 4.33, p<0.05. There was also an interaction between session and location, F (1, 36) = 8.23, p < .05. For completeness, we also report the treatment effect sizes across the two sessions where the P3a maximum has been reported to be centred (e.g. [16]). For session 1, the effects were *d* = 0.49 FZ and 0.51 CZ. For session 2, the effects were *d* = 0.56 FZ and 0.54 CZ. Analyses of the P3b component, revealed main effects of location, F (1, 35) = 9.54, p < .001, and an interaction between session and location, F (1, 35) = 6.1, p < .05. The treatment effect sizes between conditions where the P3b maximum has been reported to be centred were *d* = 0.48 and *d* = 0.28 for the central (Cz) and parietal (Pz) locations for session one For session two, the effect sizes were *d* = 0.19 and *d* = 0.16 for the central (Cz) and frontal (Pz) locations. The mean amplitude data are displayed in Table 1.

**Table 1:** The mean amplitude (uV) for the P3a (Fz and Cz scalp sites) and P3b (Cz and Pz scalp sites) across groups (control vs. rosemary) and session.

		Control Group		Rosemary Group	
		Session		Session	
		1	2	1	2
**P3a ERP**	Fz	2.95 (2.94)	0.46 (3.18)	4.67 (3.96)	2.52 (4.04)
	Cz	5.41 (3.56)	4.17 (2.52)	7.20 (3.40)	5.68 (3.10)
**P3b ERP**	Cz	2.10 (2.80)	2.49 (3.37)	3.96 (4.75)	3.24 (4.43)
	Pz	4.31 (3.50)	5.51 (3.20)	5.33 (3.90)	6.09 (3.93)

In the secondary analysis, a series of Pearson’s correlations were performed on the abovementioned data and after consideration of the scalp distributions evident in Fig 2. To determine whether the magnitude of the P3a and P3b engagement are associated with behavioural performance (Hits and R.T.). The correlations are displayed in Table 2 alongside the Fisher r-to-z transformation to test the significant difference in the relationships between control and rosemary treatment groups.

**Table 2:** Pearson’s correlations between behaviour (Hits and R.T.) and mean amplitude at P3a (Frontal; Central) and P3b (Central; Parietal) selected sites across two blocks (Electrode/Block).

Novel Stimuli (P3a Component)
F3/1 Fz/1 F4/1 C3/1 Cz/1 C4/1 F3/2 Fz/2 F4/2 C3/2 Cz/2 C4/2 Hits R.T.
Controls
Hits -.141 -.167 -.013 -.203 -.507* -.150 -.540* -.514* -.201 -.480* -.552* -.192 1 .850**
R.T. -.031 -.090 .056 -.308 -.516* -.251 -.550* -.478* -.235 -.554* -.473* -.296 .850** 1
Rosemary
Hits .355 .100 .085 .305 -.014 .053 .082 .040 .093 .031 -.207 -.165 1 .505*
R.T. .239 .308 .194 .659** .570** .663** -.087 -.043 .085 .197 .125 .212 .505* 1
Z-Hits 1.53 1.93$ 1.71 1.55 1.15 1.96$
Z- R.T. 3.11** 3.42** 2.96** 1.49 1.34 2.31* 1.79 1.96$
Targets Stimuli (P3b Component)
C3/1 Cz/1 C4/1 P3/1 Pz/1 P4/1 C3/2 Cz/2 C4/2 P3/2 Pz/2 P4/2 Hits R.T.
Controls
Hits -.105 .209 -.158 -.379 -.420* -.193 .278 .500* .380 -.559* -.438* -.319 1 .850**
R.T. -.238 .282 -.191 -.468* -.423* -.307 .213 .598** .354 -.608** -.345 -.393 .850** 1
Rosemary
Hits .054 .104 -.213 -.029 .050 -.311 -.009 -.042 -.413* -.082 -.046 -.419* 1 .505*
R.T. .485* .510* .220 .086 .267 -.108 .499* .391* -.001 .192 .280 -.238 .505* 1
Z- Hits 1.4 1.66 2.36* 1.54 1.19 0.33 1.96$
Z- R.T. 2.17* 0.77 1.67 2.03* 0.93 0.78 2.53* 1.96$

Note. $ p = .05, * p < .05, ** p < .01 for correlation and Z (Fisher’s r to z transformations were applied to significant correlations between Hits or R.T. and amplitude at the selected electrodes).

## Discussion

The primary aim of the current pilot work was to use the P3a and P3b components to probe the specificity of the rosemary’s attention-enhancing properties. Polich (e.g. [16]) outlined the P3a and P3b as the separable process of attentional capture and task focus necessary for sustained attention and effective completion of the task at hand. It was hypothesized that if rosemary enhances attention, in general, the treatment group would have higher amplitude P3a and P3b. The study also explored the possibility that the benefit of rosemary is restricted to the allocation of attention to task-related information only, and the processing of distracting information would continue to be inhibited irrespective of treatment. A further reduction in the P3a in the treatment group would suggest enhanced attentional control and inhibition of task-irrelevant information. Facilitation was observed for the P3a (effect size *d* = 0.50) and arguably P3b (effect size *d* = 0.48) component (session 1 only). These findings are consistent with the suggestion that rosemary has a global impact on sustained attention, enabling greater awareness of stimuli irrespective of relevance.

Consider first the analyses of the behavioural data. The simplicity of the task and equivalent performance in terms of hits and response times were anticipated and allowed the focus to be on the ERP brain responses. Indeed, comparable performance is desirable since differences between conditions may confound the interpretation of the brain imaging data. The P3a component has a frontal lobe and anterior cingulate origin (e.g. [17]) and has been associated with dopaminergic activity. In addition, clinical work has been informative with patients suffering left pre-frontal cortex damage having attenuated P3a, accompanied by difficulties with bottom-up orientation of attention to novel task-irrelevant information. Based on this information alone, the significant treatment effect and consistent across sessions medium effect sizes at fronto-central scalp locations, points to enhanced novelty processing. Using our model of sustained attention, all information in the environment became more accessible after rosemary ingestion regardless of relevance. Rosemary ingestion enabled the deployment of cognitive resources to task-irrelevant information at no cost to target task-relevant processing during discrimination. Under certain conditions, an increased P3a should not be seen negatively with the deeper processing of all information. Overall, the P3a data intimates more considerable attention to the novel stimuli and suggests deeper general sensory stimuli processing. Although ADHD work, for example, suggests processing information in this manner is detrimental, a processing style where more thorough and global consideration of stimulus could be beneficial (e.g. mindfulness; creativity).

Regarding the P3b component, the data should be treated with caution as they are less reliable. The medium effect was restricted to session one. However, when viewing Fig 2, an increased magnitude P3b is mainly seen at central scalp locations. Numerous brain regions have been associated with the P3b generation, but there is consensus regarding key temporal-parietal networks. Moreover, the noradrenergic (possible glutamatergic) neurotransmitter system involvement has been proposed rather than principally dopaminergic activity seen for the P3a. The key feature of the P3b is the link to the allocation of attention to task-relevant information and is reduced, for example, in dual-task situations. Similarly, facilitative effects are also seen in the long-term episodic memory domain. For example, successful memory retrieval of stimuli predicts the increased P3b amplitude at encoding [33]. The rosemary cognitive facilitation data and the P3b component converges with our knowledge of behavioural work relating to memory functioning. For example, Moss and colleagues [10] found facilitation on a serial subtractions task after rosemary aroma exposure. The cognitive operations engaged during the serial subtraction task mimic those recruited during oddball task completion. There is a need for sustained focus and concentration during serial subtractions while updating information in working memory. Similarly, during the 3-stimulus oddball task, the focus is required to process the frequent non-targets and accompanying triggering of memory updating in response to the presentation of a rare target stimulus. Under such conditions, rosemary facilitates this focus and control within working memory.

The correlation data provided an additional level of explanation when considering the therapeutic impacts when including multiple measurements of behavioural (e.g. Reaction times and accuracy) and electrophysiology. Indeed, the joint consideration of electrophysiological and behavioural measures has been championed [34]. Thus, examining the commonalities of relationships among multiple parameters across treatment could best explain the therapeutic effects. Given the large set of correlations (Table 2), we restrict our discussion to significant correlations between groups and when this difference is consistent across electrodes. That said, we must still be cautious in interpreting this secondary analysis due to alpha-level inflation errors. Further, it is worthwhile noting the wider consideration of the scalp sites was based on the observed scalp distributions displayed in Fig 2.For the P3a, this occurred at central sites (C3,Cz,C4) in session one. First, it is worth considering the ‘typical’ pattern of behaviour in the control condition. A consistent pattern was seen across all electrodes where the P3a is centred for hits and reaction time. The traditional view of the P3a is that it serves as a useful index of distraction. Under this view, lower P3a amplitudes indicated less capture and, therefore, more accurate responding. The evidence of less distraction was also in line with increased reaction time and perhaps more performance monitoring and careful responding. There is less effective discrimination and poorer and quicker responses in those individuals where the novel stimuli capture attention. This ‘typical’ behaviour invites the question of whether rosemary modifies this pattern of associations. The findings were limited to response times in the rosemary condition. Indeed, an increase for targets was related to a rise in P3a amplitude. This likely occurred due to more in-depth processing of stimuli irrespective of relevance. Perhaps the increased magnitude of the P3a in the rosemary condition indicates processing the distractors while contemplating the response to the task-relevant targets. Unlike the control condition, the increase in distraction indexed by an increase in the P3a did not impact on accurate responding. Rosemary gave rise to increased cognitive resources to process all information irrespective of relevance. So, in this case, a higher magnitude P3a is not unfavourably as there was no impact on task-relevant processing. The pattern of association is unlike ADHD with known behavioural problems processing task-irrelevant information (e.g. [23]), increased P3a amplitude and subsequent impairment of task-relevant information (e.g. a decrease in the P3b). In a similar vein to the main ERP findings, the pattern of associations between behaviour and ERPs is less clear for the P3b component. However, these data help interpret the abovementioned P3a data. A higher magnitude P3b, related to a greater allocation of attention to task-relevant information, was related to slower response times. Presumably, a great awareness of all stimuli for the rosemary group led to increased response time to maintain performance while attending to all.

Considering the psychopharmacological underpinnings, active compounds have been proposed as putative drivers of cognitive enhancement. For example, Moss and Oliver [10] linked the cognitive facilitation processes with serum levels of 1,8-cineole, a compound that inhibits acetylcholinesterase [1]. Their key findings suggest that 1, 8-cineole has a particularly positive influence when certain conditions are met. First, the cognitive processes engaged should include attentional control and working memory elements. Second, cognitive demand seems critical, with a more significant effect for tasks with medium cognitive demand (serial threes). In the present study, we have argued our P3 paradigm effectively measure different aspects of attention similar to earlier behavioural work in the area. Therefore, it would be interesting in future studies to directly investigate combined behavioural and EEG measures and how they related to plasma 1, 8 –cineole and other chemical constituents, which may underlie the effects. We also need to ask how our neurobiology knowledge of the P3 indices contributes more generally to uncovering psychopharmacological impacts. For instance, the P3a has links to attention reported here, emotion processing and dopaminergic activity (see [35] for discussion). Whereas the P3b is associated with memory and norepinephrine parietal activity [15]. Possessing this knowledge has been useful elsewhere when proposing neurotransmitter and psychopharmacological interactions (e.g. Glucose: [22]).

## Conclusions

In summary, previous research has demonstrated the facilitation of cognition after exposure to the aroma of rosemary and ingestion of rosemary water (e.g. [36]). The present work used the precision of ERPs to probe for the first time the different aspects of sustained attention that may benefit. The absence of a selective effect of ERPs measured suggests rosemary provides additional attentional resources to process all events we encounter, irrespective of relevance. Much like our other pharmacological and nutritional, behavioural impact studies and elsewhere, future experiments should include different doses and/or an additional herbal extract to truly determine the influence on ERP markers of attention. Regardless, it is clear that ERP subcomponents are useful neuropsychological tools to use alongside behavioural measurement to examine selective facilitation and the therapeutic effect of herbal compounds.

## Abbreviations

ERP: Event-related potentials
EEG: Electroencephalography
ANS: Autonomic nervous system
ADHD: Attention deficit hyperactivity disorder
ANOVA: Analysis of variance
